# A hospital care coordination team intervention for patients with multimorbidity: A practice-based, participatory pilot study

**DOI:** 10.1177/17423953231196611

**Published:** 2023-09-06

**Authors:** Marlies Verhoeff, Janke F. de Groot, Hanneke Peters-Siskens, Erik van Kan, Yolande Vermeeren, Barbara C. van Munster

**Affiliations:** 1 Department of Internal Medicine, University Center of Geriatric Medicine, University Medical Center Groningen, Groningen, The Netherlands; 2 Knowledge Institute of the Federation of Medical Specialists, Utrecht, The Netherlands; 3 Department of Internal Medicine, Gelre Hospitals, Apeldoorn/Zutphen, The Netherlands; 4 School of Health Studies, HAN University of Applied Sciences, Arnhem/Nijmegen, The Netherlands; 5 Department of Clinical Pharmacy, Gelre Hospitals, Apeldoorn/Zutphen, The Netherlands

**Keywords:** Multimorbidity, hospital care, care coordination, fragmented care, care coordination intervention

## Abstract

**Objectives:**

This study aims to develop and pilot a hospital care coordination team intervention for patients with multimorbidity and identify key uncertainties

**Methods:**

Practice-based, participatory pilot study with mixed methods in a middle-large teaching hospital. We included adult patients who had visited seven or more outpatient specialist clinics in 2018. The intervention consisted of an intake, a comprehensive review by a dedicated care coordination team, a consultation to discuss results and two follow-up appointments. We collected both quantitative and qualitative data.

**Results:**

Out of 131 invited patients, 28 participants received the intake and comprehensive review. The intervention resulted in mixed outputs and short-term outcomes. Among the 28 participants, 21 received recommendations for at least two out of three categories (medication, involved medical specialists, other). Patients’ experienced effects ranged from no to very large effects. Key uncertainties were how to identify patients with a need for care coordination and the minimum of required data that can be collected during regular clinical care with feasible effort.

**Discussion:**

Recruitment and selection for hospital care coordination should be refined to include patients with multimorbidity who might benefit most. Outcomes of research and clinical care should align and first focus on evaluating the results of care coordination before evaluating health-related outcomes.

## Introduction

Patients with multimorbidity, generally defined as two or more chronic conditions, have lower health-related quality of life, more functional disabilities and use more healthcare compared to patients with a single chronic condition.^1–5^ Current hospital systems, however, are organized around single conditions and separate medical specialties, which results in fragmentation of care for patients with multimorbidity.^6,7^ Fragmented care delivery by multiple healthcare providers, with low coordination and continuity of care, is associated with overuse of diagnostic procedures and treatment, adverse drug events, complications and higher costs.^8–10^ To meet the growing demand and avoid potentially preventable consequences of fragmentation of care, healthcare policy organizations urge that healthcare delivery for multimorbidity should transform to so-called integrated care.^11–13^ Nevertheless, previous research on integrated care only suggests limited effects on patient outcomes such as quality of life and mortality.^11,12^

In our opinion, this is partly due to the choice of outcome measures and the classical approach of research that does not take the complexity of multimorbidity and the interventions into account. Implementing and measuring the efficacy of integrated care interventions are complex due to:

- the number of interacting components (diseases, treatments, patient and multiple care providers);
- the number and difficulty of behaviours required by those delivering or receiving the intervention (accepting coordination of care, deviating from guideline recommendations, working differently or receiving other care than usual);
- the number and variability of disease-related, patient- and healthcare-related outcomes.

The Framework for Developing and Evaluating Complex Interventions offers guidance for the development and evaluation of complex interventions, and the Logical Framework Approach can make implicit knowledge of a complex intervention explicit.^14^ This explicit knowledge regarding the activities, output and outcomes of the intervention can be used to better understand how, why or which parts are effective, using practice-based evaluations.^15^ When using a Logical Framework Approach, researchers look at direct outputs and related outcomes to learn lessons about the efficacy and explaining mechanisms.^16^

The aim of this practice-based, participatory pilot study with mixed methods was to use the described frameworks to develop and pilot a complex intervention with a hospital-based care coordination team for patients with multimorbidity and identify key uncertainties. The goal of the intervention was to achieve coordinated hospital-level outpatient care, tailored to the individual needs and preferences of patients with multimorbidity.

## Methods

The Framework for Developing and Evaluating Complex Interventions describes the basic elements for the development and evaluation of complex interventions: development, feasibility and piloting, evaluation and implementation.^14^ We developed a hospital-based care coordination intervention and programme theory based on previous research and clinical expertise. Next, we piloted the intervention to evaluate the design, identify key uncertainties and assess whether refinement of our programme theory and intervention was necessary. As an updated version of the framework was published when this article was being written, we followed the updated framework for the evaluation and reporting.^17^

### Programme theory and development of the intervention

**Figure 1.**
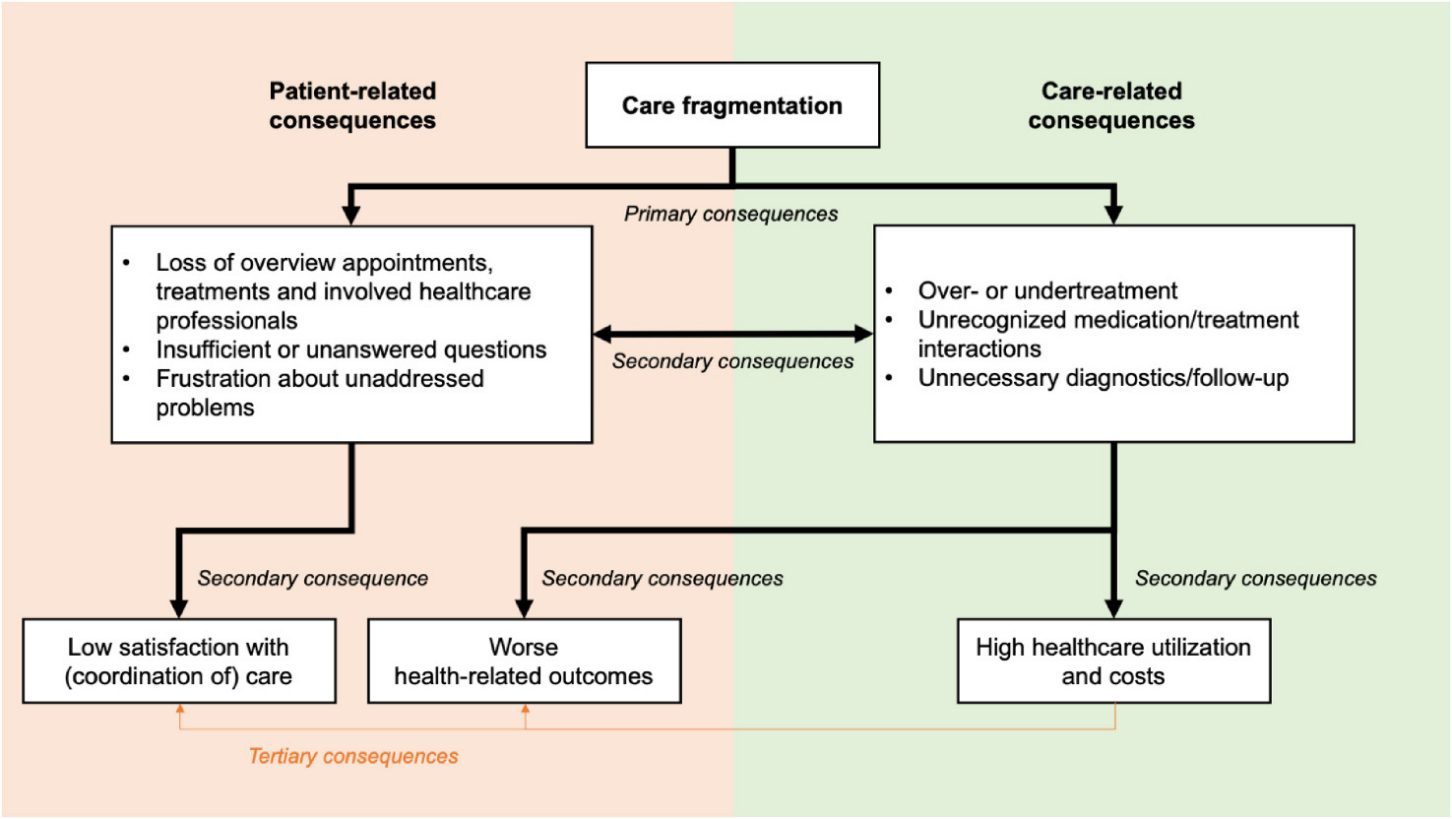
Causal chain of care fragmentation. The potential primary and secondary patient- and care-related consequences of care fragmentation. Some secondary consequences can result in tertiary consequences as well.

Based on current literature, a qualitative study, and a case report, we identified fragmentation of hospital care as the main problem for patients with multimorbidity.^7,8,18–20^ Care fragmentation can result in a loss of overview of appointments, treatments and involved healthcare professionals and lead to insufficient or unanswered questions and frustration about unaddressed problems.^21,22^ Care fragmentation is also associated with over- or undertreatment, unrecognized treatment interactions and adverse events and unnecessary diagnostics/follow-up.^8,9,18–20^ These potential primary consequences of care fragmentation can lead to secondary, often more long-term, consequences such as worse health-related outcomes, low satisfaction with care and high healthcare utilization and costs. Figure 1 depicts this causal chain of care fragmentation, with the primary, secondary and tertiary consequences.

The underlying theory of our complex intervention was the hypothesis that creating an overview of hospital care and medication and giving recommendations for tailoring and coordination of hospital care would help improve the primary and secondary consequences of care fragmentation as described in Figure 1. Another hypothesis was that a care coordination intervention would be most valuable for patients who see many different medical specialists, but who do not have an obvious diagnostic/treatment process that is likely to entail care coordination. This would exclude patients with oncological diagnostics/treatment, patients receiving dialysis, patients consulting the geriatrics outpatient clinic or patients in a terminal phase of life.

**Figure 2.**
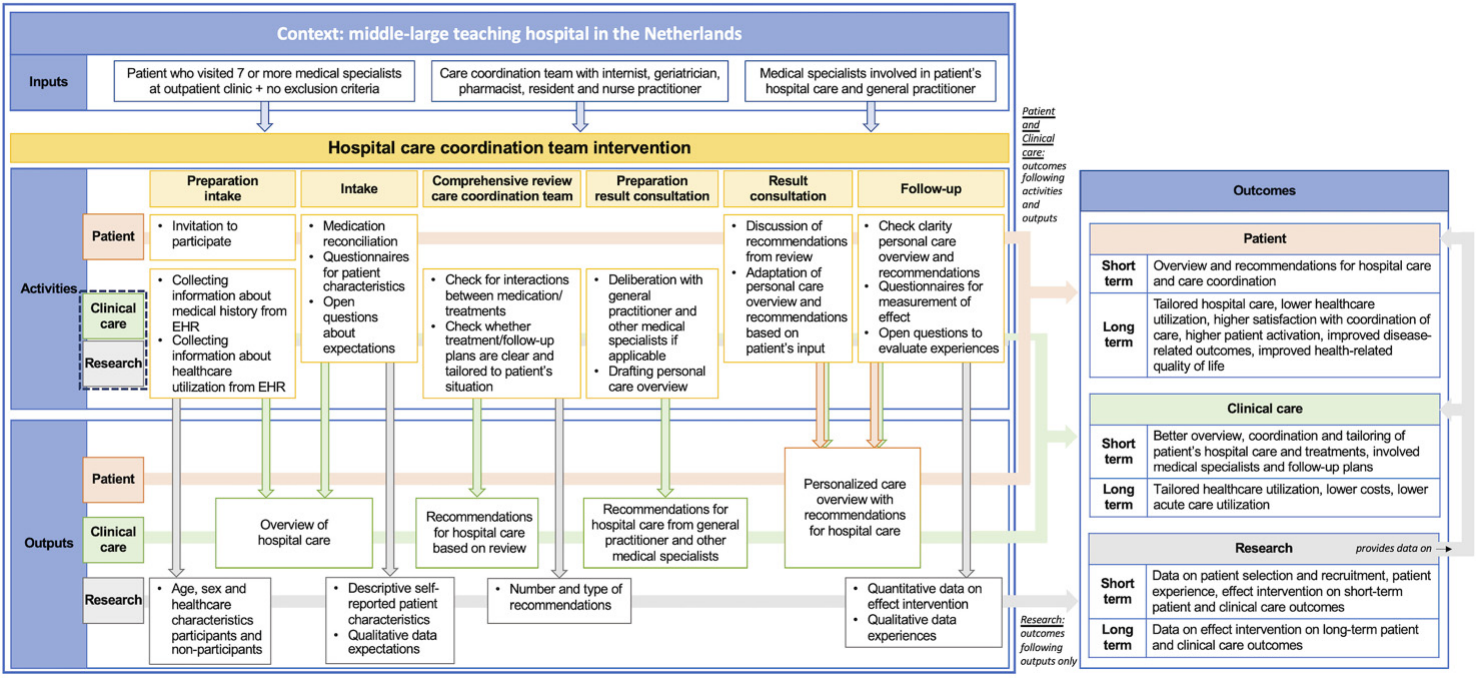
Programme theory for a hospital care coordination team intervention with the patient-related, care-related and research-related activities, outputs and outcomes.

We based the intervention's design on the general workflow for a comprehensive geriatric assessment in our hospital. We applied the same structure: elaborate intake, comprehensive review by the care coordination team (instead of diagnostics), consultation to discuss recommendations from the review and follow-up consultation. To develop the programme theory and describe the intervention, we used the Logical Framework Approach.^15^ For every component, we described the activities, outputs and outcomes for the patient, clinical care and research (this pilot study). The final programme theory and intervention are depicted in Figure 2.

### Ethical approval and sample size for the pilot study

The Daily Board of the Medical Ethics Committee X (190604 HOCOM) and the institutional review board X (number 2019_26) reviewed and ethically approved this pilot study. Written informed consent was obtained from all participants. Participants were informed that their cases would be discussed with the care coordination team. All collected data was coded and pseudonymized for analysis.

We did not execute a formal sample size calculation. We aimed to include 50 participants because we estimated that this would provide sufficient information for evaluation.

### Context of the intervention

The study was conducted over a 9-month period at the internal medicine outpatient clinic of a middle-large teaching hospital. Medical specialists and general practitioners were informed via their newsletters about the pilot study. The medical hospital staff was asked for approval to approach their patients. The pilot was presented in a regional general practitioners’ meeting and for the internists and residents at the internal medicine outpatient clinic where the intervention would take place.

#### Patients

We included adults (18 years or older) who had visited seven or more outpatient specialist clinics at the hospital in 2018. We expected these patients to have a high risk of experiencing consequences of care fragmentation due to the high number of outpatient specialist clinics they visited. An analysis of the hospital's electronic health records showed that a cut-off value of seven would result in 461 potential participants. We repeatedly selected a random sample of 50 of the 461 potential participants using the sample (base) function in R.^23,24^ Patients were excluded if:

- they were actively treated by a geriatrician, receiving dialysis or oncological treatment;
- they were in a terminal phase (live expectation < 6 months) according to the electronic health record (EHR);
- there were less than two future appointments;
- there was another reason why participation was not deemed feasible.

If potential participants did not meet the exclusion criteria, they were sent a letter inviting them to participate, followed by a phone call by a research assistant after 1–2 weeks to plan the intake appointment if they were willing to participate.

*Care coordination team*. The care coordination team consisted of a nurse practitioner and a clinical research physician, who also conducted all appointments, executed the research activities and created the personal care overview. For the comprehensive review, they were joined by an internist and a geriatrician, both hospital generalists who have experience with multimorbidity, and a hospital pharmacist, who has expertise to advise on polypharmacy.

### The hospital care coordination team intervention: activities, outputs and outcomes

For this pilot study, we decided to focus on the direct outputs and short-term outcomes to evaluate the intervention's design and its effect on the primary consequences of care fragmentation (as described in Figure 1). Supplemental file 1 and Supplemental file 2 show an elaborate description of the intervention and an example of a personalized care overview, respectively.

*Clinical care- and research-related activities, outputs and outcomes*. The clinical research physician and nurse practitioner conducted all activities. Clinical care- and research-related activities were connected and overlapping but were considered different types of activities with different outputs. The clinical care-related activities are activities that in other settings can be a part of regular clinical care. These activities were aimed at:

- creating an overview of hospital care and a patient's situation, including drafting a personalized care overview;
- producing recommendations for hospital care based on the review by the care coordination team and any deliberation with the general practitioner and/or involved medical specialists.

The research-related activities were activities to collect both quantitative and qualitative data for the evaluation of the intervention's design and the pilot study. We collected data at intake to describe participant's characteristics and their expectations, during the review process about the recommendations and at follow-up about the experienced effect.

During preparation, information about the medical history, medication and involved hospital physicians was collected (clinical care and research). The intake included a medication reconciliation and preliminary review, and discussion of the intake questionnaire plus any other issues identified during preparation (clinical care and research). During the comprehensive review, the hospital care coordination team checked indications and potential treatment interactions and adverse events. The team also checked whether treatment/follow-up plans were clear and tailored to the patient's situation. If any treatment interactions and adverse events, unclear indications or unclear treatment/follow-up plans were recognized, the team would formulate recommendations. If necessary, there was deliberation with the general practitioner and/or involved medical specialists after the meeting. The recommendations were recorded (clinical care) and afterwards quantified by type of recommendation (research).

After 4 weeks, when the comprehensive review had been completed, the participant returned for the result consultation. The recommendations were discussed with the participant (clinical care). They received a personalized care overview including their medical history, medication, involved healthcare professionals and the recommendations (clinical care). If the participant provided new input, the personal care overview was adapted if necessary.

At follow-up, another 4 to 6 weeks later, the nurse practitioner checked by phone whether the participant had any questions about the personalized care overview (clinical care). At the 3-month follow-up, the participants answered questions about their experiences with the intervention (qualitative data on short-term outcomes) and filled out a follow-up questionnaire (quantitative data on long-term outcomes). The data collected at the 3-month follow-up were for research purposes.

The short-term outcomes for clinical care were a better overview, coordination and tailoring of patient's hospital care and treatments, involved medical specialists and follow-up plans. The short-term outcomes for research were data to evaluate the intervention's design and the pilot study's short-term outcomes: patient characteristics, patient experiences and the short-term patient- and clinical care-related outcomes.

*Activities, outputs and outcomes for patients*. The aim of the patient activities was to create a personalized care overview for the participant and provide recommendations for hospital care and care coordination. Short-term outcomes for the participant were overview and personal recommendations for their hospital care and care coordination.

### Evaluation and identification of key uncertainties

To evaluate and refine the programme theory and intervention, we used the collected data for research plus a focus group evaluation with the care coordination team. We evaluated the effect on short-term outcomes for patient, clinical care and research and identified key uncertainties.

For the evaluation of patient-related short-term outcomes, we used the qualitative data on participant's expectations and experiences of the intervention. For the evaluation of care- and research-related short-term outcomes, we used the quantitative and qualitative data collected at intake and review. The focus group evaluation with the care coordination team was chaired by an independent senior researcher (JdG) who had not been involved in the design and piloting of the intervention. During the focus group evaluation, the team reflected on their experiences with the intervention and on the short-term outcomes for patient, clinical care and research, in order to identify key uncertainties.

### Adjustment and early termination due to COVID-19

The COVID-19 outbreak led to early termination of the study, because the hospital was no longer able to keep up with regular care outside COVID-19. We were forced to adjust the results of consultations and follow-ups. All result consultations and follow-ups were done by phone instead of at the outpatient clinic. Because the evaluation of long-term outcomes with the follow-up questionnaire was not the objective of the pilot, we only asked questions about the participant's experiences of the intervention at follow-up.

## Results

### Patient selection and recruitment

**Figure 3.**
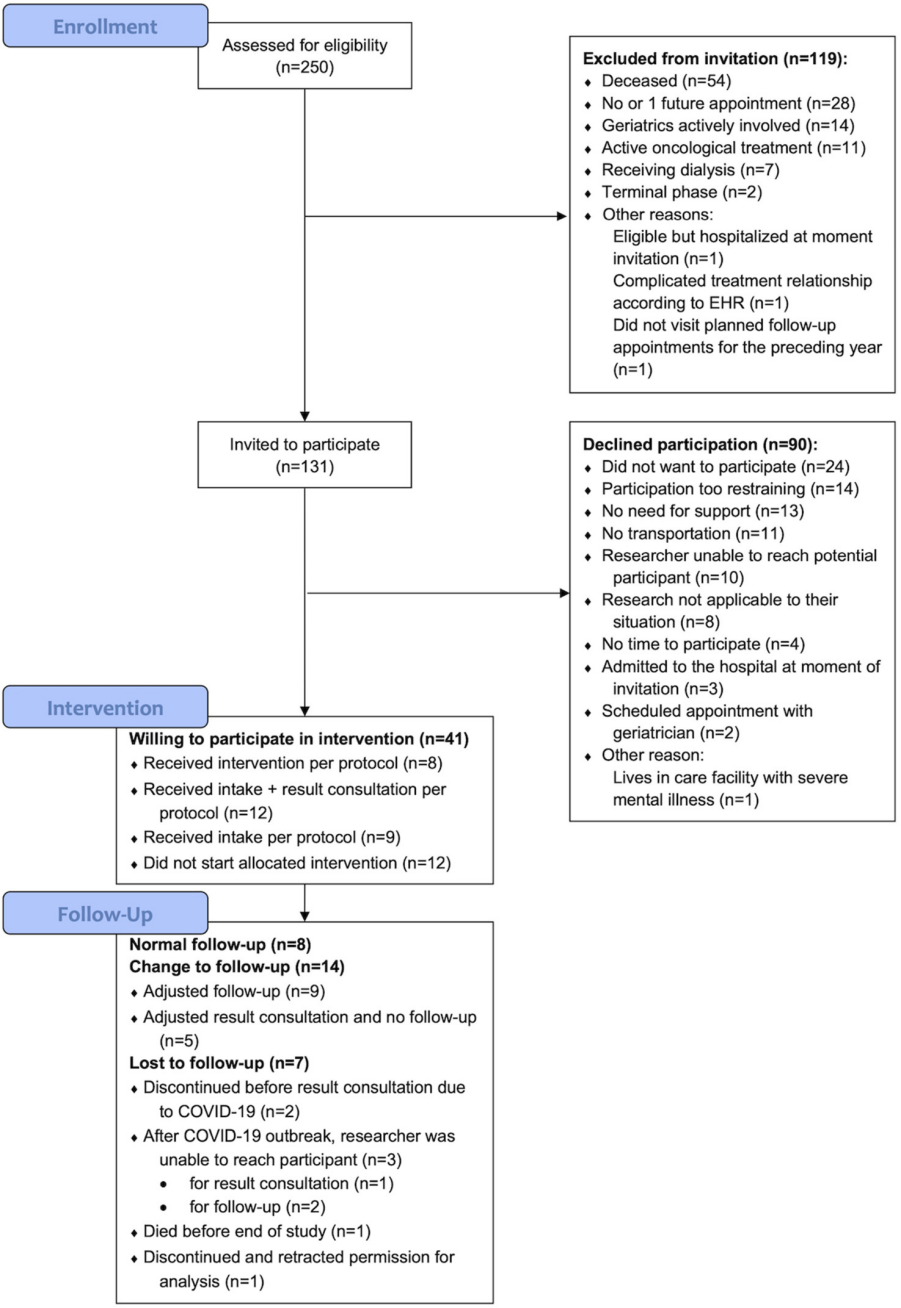
Flow chart of the patient selection and recruitment process.

We assessed 250 (5 × 50) randomly selected patients for eligibility. We excluded 119 patients, 54 of these patients (45,4%) were deceased at the time of assessment and 28 patients (23,5%) had less than two scheduled appointments. A flow chart of the complete patient selection and recruitment process is shown in Figure 3.

Eventually, 131 patients were invited to participate. In the group with patients who declined participation (n = 90), 14 patients indicated that participation would be too restraining, and 11 patients could not participate because they did not have transportation. Additionally, 13 patients indicated that they had no need for support, and eight patients responded that the research was not applicable to their situation. There were no significant differences in age, sex or number of hospital physicians visited in 2018 between the group who was willing to participate (*n* = 41) and the group who declined to participate (*n* = 90) (see Supplemental file 3).

During the 9-month period, we recruited 41 patients who were willing to participate. In the end, 28 participants received the intake before the COVID-19 outbreak. One other participant started the intervention but discontinued and retracted permission for analysis, so these results were removed from the database. Eight participants completed the 3-month follow-up visit at the outpatient clinic before the COVID-19 outbreak. A total of 17 participants completed the result consultation in real life and/or adjusted follow-up by phone, and three participants received the result consultation by phone but could not be reached for an adjusted follow-up. Baseline characteristics at intake (*n* = 28) are shown in Table 1.

**Table 1.** Baseline characteristics at intake (n = 28).

Characteristic	
Demographic	
Age, median (IQR)	72.3 (63.2–77.2)
Sex, *n*(%) female	17 (61)
Relationship status, *n* (%)	
*Married or living together with partner*	22 (79)
*With partner but not living together*	1 (4)
*Without partner*	5 (18)
Work situation, *n* (%)	
*Working fulltime/parttime*	1 (4)
*Disability leave*	8 (29)
*Retired*	18 (64)
*Unemployed*	1 (4)
Highest education level, *n* (%)	
*Primary school*	2 (7)
*High school*	13 (46)
*University*	12 (43)
*Unknown*	1 (4)
Living situation, *n* (%)	
* Independent*	28 (100)
Home care, *n* (%)	10 (36)
*Assistance with bathing*	5 (18)
*Assistance with getting dressed*	3 (11)
*Assistance with household tasks*	5 (18)
Informal care, *n* (%)	
*Receiver/giver*	15 (54) / 9 (32)
*High level of Caregiver Strain Index, n(%)*	3 (11)
Functional status	
KATZ-ADL 6 score	
*0 points*	17 (61)
*1 point*	4 (14)
*2 points*	6 (21)
*3 points*	1 (4)
Walking aid, n (%)	17 (61)
Other medical aid, n (%)	16 (57)
Reported at least one fall in last 6 months, n (%)	10 (36)
Health status	
HRQOL (EQ5D-5L index), median (IQR)	0.73 (0.58–0.81)
EQ5D-VAS, median (IQR)	63 (50–71)
Symptoms, mean (SD)	10 (5)
Patients with depressive symptoms (PHQ ≥2), n (%)	5 (18)
Positive Six Item Cognitive Impairment Test (6-CIT ≥8), n(%)	4 (14)
Patient activation, health literacy and satisfaction with care	
Number of patients with insufficient health literacy (SBS-Q), n (%)	2 (7)
Patient activation (PAM-13), n (%)	
Level 1 (believes active role important)	4 (14)
Level 2 (confidence and knowledge to take action)	8 (29)
Level 3 (taking action)	13 (46)
Level 4 (staying the course under stress)	4 (14)
Patient satisfaction with care (PACIC), median (IQR)	2.8 (2.1–3.2)
Healthcare utilization in 12 months pre-intake (from EHR)	
Diagnoses that were treated in the hospital, mean (SD)	7 (3)
Number of outpatient visits, median (IQR)	20 (16–24)
Number of ED visits, median (IQR)	0 (0–1)
Number of hospitalizations, median (IQR)	0 (0–1)
Number of hospitalization days, median (IQR)	0 (0–3)
Involved medical specialties in 2018, n (%)	
Internist	21 (75)
Cardiologist	20 (71)
Pulmonologist	20 (71)
Ophthalmologist	19 (68)
Surgeon	16 (57)
Orthopedic surgeon	13 (46)
Anesthesiologist	12 (43)
Neurologist	12 (43)
Ear-nose-throat	11 (39)
Gastroenterologist	11 (39)
Rheumatologist	11 (39)
Dermatologist	10 (36)
Urologist	9 (32)
Dental surgeon	8 (29)
Gynecologist	7 (25)
Plastic surgeon	4 (14)
Neurosurgeon	1 (4)
Physiatrist	1 (4)
Follow-up plans	
Follow-up appointment planned, median (IQR)	3 (2–4)
Appointment if patients has complaints, median (IQR)	1 (0–1)
Follow-up ended	3 (2–3)
Unclear	0 (0–1)

### Recommendations from review by the care coordination team

Table 2 gives an overview of participants, recommendations and the experienced effects of the intervention. Out of 28 participants, 21 received extensive recommendations, e.g. for at least two out of three categories (medication, involved medical specialists, other). One participant did not receive any recommendations. With one participant, we discussed unclear results from previous consultations. Five participants received recommendations for small adjustments in one or two medications (stop medication without indication or change of one medication due to (risk of) adverse events); they did not receive other recommendations. One participant who received care coordination recommendations did not receive any other recommendations.

**Table 2.** Overview of participants, recommendations, and experienced effects of the intervention (n = 28).

Ref no.	Sex	Age	Involved medical specialties year prior	Planned appointments	QoL (EQ5D-VAS)	Advice for ≥2 categories (yes/no)	Medication*			Involved medical specialties*			Other recommendations*	Effect intervention** (Likert scale 1–5) on:	
							Change (*n*)	Stop (*n*)	Start (*n*)	Change (*n*)	Stop (*n*)	Start (*n*)		Most important regarding diseases	Most important regarding hospital care
1	Female	60	7	3	90	Yes	1	1	0	-	-	-	Care coordination, lifestyle	1	1
2	Male	77	10	3	60	Yes	1	0	0	-	-	-	-	1	1
3	Female	75	8	4	65	Yes	**3**	0	0	1	0	0	Lifestyle	3	1
4	Male	49	7	4	70	Yes	0	**5**	0	1	0	1	-	1	1
5	Male	82	8	4	65	No	0	**2**	0	-	-	-	-	4	1
6	Male	77	8	6	50	Yes	**2**	0	0	-	-	-	Order of treatment/prioritizing	3	3
7	Female	76	7	2	60	No	-	-	-	-	-	-	Discussion of unclear previous results/consultations	1	1
8	Female	50	8	3	50	No	**2**	**3**	0	-	-	-	-	1	1
9	Male	87	3	3	70	Yes	0	**4**	0	-	-	-	Advice to draft a plan for pain control	1	1
10	Female	77	7	4	75	Yes	1	**3**	0	1	0	0	Care coordination	4	2
11	Female	78	7	2	50	No	-	-	-	-	-	-	-	No follow-up	No follow-up
12	Male	60	7	5	45	No	-	-	-	-	-	-	Care coordination, conversation about advance care planning/resuscitation preferences should be considered	5	5
13	Female	82	7	3	75	Yes	0	1	0	1	1	0	-	2	2
14	Female	73	8	2	50	Yes	**2**	1	1	**2**	0	0	Psychological support (to be considered)/advice on coping/stress etc.	3	3
15	Female	66	7	3	90	No	0	1	0	-	-	-	-	No follow-up	No follow-up
16	Male	74	9	2	70	Yes	1	1	1	-	-	-	Lifestyle	No follow-up	No follow-up
17	Male	62	7	2	60	Yes	0	1	0	-	-	-	Lifestyle	No follow-up	No follow-up
18	Female	60	8	2	50	Yes	1	1	**2**	1	0	1	-	4	3
19	Male	72	8	3	70	Yes	**2**	1	**2**	-	-	-	Order of treatment/prioritizing, non-medication advice about specific diseases	3	1
20	Female	87	8	3	50	Yes	**3**	1	0	-	-	-	Conversation about advance care planning/resuscitation preferences should be considered	No follow-up	No follow-up
21	Female	62	7	3	40	No	1	0	0	-	-	-	-	2	5
22	Female	68	7	2	40	Yes	0	**2**	1	-	-	-	Care coordination, psychological support (to be considered)/advice on coping/stress etc., Instruction about medication intake	No follow-up	No follow-up
23	Male	68	7	3	20	Yes	0	**3**	1	-	-	-	Care coordination	No follow-up	No follow-up
24	Female	80	8	6	45	Yes	**2**	0	0	**2**	0	0	Care coordination	4	1
25	Female	67	8	6	75	Yes	**3**	1	0	1	1	0	Instruction about medication intake	No follow-up	No follow-up
26	Female	69	7	3	70	Yes	0	1	0	1	1	0	Discussion of unclear previous results/consultations	No follow-up	No follow-up
27	Male	73	7	2	75	Yes	0	1	1	-	-	-	Care coordination	No follow-up	No follow-up
28	Female	64	7	4	80	Yes	**2**	0	0	1	0	0	-	No follow-up	No follow-up
**Summary of recommendations:**						21 patients	26 patients with recommendations			10 patients with recommendations			18 patients with recommendations		

*Recommendations: (Color version available in Online) green color shows that the team gave one or more recommendation(s); darker green corresponds with recommendations for more than one medication/medical specialty. **Effect intervention: green color shows that the patient gave a higher score on the Likert scale. Orange and red color correspond with lower scores on the Likert scale.

### Participants’ experiences

The answers to the three open questions at intake are shown in Supplemental file 4. At follow-up, five out of seventeen participants reported that the intervention had a large or very large effect on what was most important to them. They all reported that participation had made them think about their care and their own health situation. One participant realized that living completely independently was not realistic anymore:

Yes, I have reached the conclusion that I cannot stay independent like this any longer. If I fall, I will not get up anymore. I have registered for supportive housing. (P10)

Other participants reported thinking more extensively about their health, its place in their lives and their contacts with physicians.

The intervention has had a large effect on me: I think more about the state of affairs. I think more about my own situation and about the contact that I have with physicians. (P5)

Lifestyle remains difficult, I try, but I have always lived an abundant life. Because of this intervention I have become more conscious that things are connected to each other, there is a holistic picture of health and I see the importance of that. (P12)

Another participant experienced participation as helpful and supportive, because of the overview and the sympathetic ear it provided. It helped to realize that ‘it was all quite manageable’ (P24)*.*

Eight out of 17 follow-up participants reported that the intervention had little or very little effect on what was most important to them regarding the treatment of their diseases.

It did not have an effect on [my complaint] but I did receive medical aids to help dress myself. (P2)

Participation has not changed my tiredness. I have put the bar less high, that gives me peace of mind. (P7)

The pain has not changed. (P8)

Twelve out of 17 follow-up participants reported very little or little effect on what they wished to see improved in the hospital care organization. Three participants reported a neutral effect. Two participants reported a very large effect:

I have experienced the effect a couple of times, with the pulmonologist, the internist and the cardiologist. They coordinated with each other. (P12)

I have become more vigilant when it comes to standing up for myself when I am at the doctor. (P21)

Eleven out of 17 participants reported that they had taken another look at the personal care overview, and five participants reported that they had used the overview. Three participants used the overview with their GP to discuss the recommendations. One participant used the overview to check details. Another participant reported taking the overview to the cardiac emergency room. The participant reported that the healthcare providers at the ER were very happy with the overview and that it was useful during the consultation. Participants who had not used the overview, said they had ‘no need for it’, ‘no reason to use it’ or ‘not thought about using it due to COVID-19’.

### Care coordination team's experiences

During the focus group session, the care coordination team reflected on their experiences with the intervention and on the short-term outcomes for patients, clinical care, and research. The key uncertainties were:

- How to identify patients with a high risk of experiencing the consequences of fragmentation of care, who might benefit most from the intervention?
  Having visited seven or more outpatient specialist clinics in the year prior does not seem specific as a selection criterium. Some patients did not seem to experience consequences of fragmented care (anymore) or did not have many medical specialists involved anymore. The patient selection and recruitment process yielded a few patients who might benefit most, when looking at the recommendations, although most patients did receive at least one recommendation. However, the care coordination team reflected that this extensive intervention would not be necessary for most patients to provide these recommendations. The selection process, and the number and extent of recommendations, also showed that high numbers of medical specialists, polypharmacy and multimorbidity are not perfect synonyms for patients with a high risk of experiencing consequences of fragmentation of care.
- What is the minimum of data that can be collected for research to evaluate the efficacy, with limited time investment and effort for patients and regular care professionals?
  It was an extensive intervention to coordinate hospital care for patients with multimorbidity. Preparation for the intake, the numerous validated questionnaires required for research purposes and creating the personal care overview by hand took up most time. The mix of aiming to change clinical care outcomes and at the same time measure the outcomes for research purposes with elaborate questionnaires resulted in an extensive process.

The main insights were:

- With thorough preparation by the research physician or nurse practitioner, the comprehensive review by a care coordination team did not take more effort than a regular multidisciplinary team meeting for the medical specialists involved.
- Beforehand, the medical specialists were apprehensive about displaying their knowledge, but eventually they thought it was a smooth and educational experience.

## Discussion

### Summary of main findings

The aim of this practice-based, participatory pilot study was twofold: (1) We developed and piloted a hospital-based care coordination team intervention that aimed to achieve coordinated hospital-level outpatient care, tailored to the patient's needs and preferences, and (2) we used practice-based, participatory research, based on the Framework for Developing and Evaluating Complex Interventions and the Logical Framework Approach, to gain more insight in key uncertainties of the intervention. We will reflect on the lessons learned.

*Lessons learned: the efficacy of the hospital care coordination intervention*. Our pilot study showed that the intake and comprehensive review by the hospital care coordination team resulted in recommendations ranging from minor recommendations for medications to more extensive recommendations regarding coordination of care, involvement of medical specialties and medication. The varying experiences of effect reported by patients also reflected the variability of efficacy. The team recognized this variability as well. The hypothesis that this type of hospital care coordination intervention would be effective for most patients visiting more than seven medical specialties was not supported by these results. We hypothesize it might be an effective intervention in a subpopulation with a high number of medical specialties who are experiencing adverse consequences of fragmented care.

Not all patients who had visited seven or more medical specialties seemed to be experiencing consequences of fragmented care or requiring support. For example, there was a group of potential participants who did not have more than one future appointment. Another group of invited patients declined participation because they did not need support. Moreover, the included patients had planned follow-ups for a median of only three medical specialties, instead of seven. That patients do not always use care or require support over a long period is in line with the research that showed that not all patients are persistently high-cost and/or high-need patients.^25–27^ Furthermore, the timing of the intervention might have been too late: 20% of the potential participants were deceased at the time of recruitment and selection. Wammes et al. (2018) reported that almost 30% of high-cost patients are in their last year of life.^28^ It is common for patients in their last year of life to visit multiple medical specialties, but healthcare professionals report barriers to the initiation of end-of-life care discussion and advance care planning.^29,30^ If patients received the intervention quickly after identification, it might help in starting advance care planning or end-of-life discussion.

Future efforts should aim to find the patients with multimorbidity who experience adverse consequences of fragmented care and how to recognize them in clinical practice. To help care professionals recognize these patients, hospitals might implement prediction models for high healthcare utilization or worse health-related outcomes or start screening for problems in care coordination.^31^

*Lessons learned: practice-based, participatory research with integration of research in clinical care*. Despite the benefits of a practice-based, participatory study design, there are also some drawbacks to the difference in the two main aims of the intervention, e.g. changing care delivery for the individual patient versus studying the efficacy of changed care delivery at an aggregated level. We learned that the extra patient activities required for research might be a barrier for patients to participate. The participants were all living independently, and only one-third experienced slight functional limitation. Possibly, patients with more severe functional limitations did not participate because the intervention meant extra care activities next to their regular care. This hypothesis was supported by the fact that a group of patients declined participation because they thought it too restraining or did not have transportation. A solution might be to include patients at the moment of a potential adverse consequence of fragmentation, for example, at an acute admission.^32^ Embedding the regular clinical care process could make it less restraining. Consequently, it might help to include more patients with functional disability or severe disease. Another solution, learned from the COVID-19 pandemic, is teleconsultation instead of outpatient clinic visits when transportation or time are limiting factors.^33^

From a healthcare professional perspective, we learned that working together in this care coordination team was not as much of a burden as expected. In fact, the healthcare professionals who participated in the care coordination team were positive about working together and learning from each other. However, the administrative burden for the research physician and nurse practitioner was high, because of the combination of research and clinical care activities and the questionnaires and forms that had to be filled out for research purposes. This administrative burden could be a barrier for healthcare professionals to participate in this intervention, as ‘time constraints’ and ‘excess paperwork’ have been mentioned before as barriers to participation in clinical research.^34^

Ideally, to ensure smooth implementation of an integrated care intervention into regular clinical care, research activities should minimally impact regular clinical care activities. Options for minimal impact are aligning clinical care delivery outcomes with research outcomes by choosing carefully which outcomes or questionnaires are truly necessary for both. In previous research, health-related quality of life was often a primary outcome measure, but it is a secondary consequence of care fragmentation, and generally not used to inform clinical care decisions.^11,12^ Outcome measures should measure the result of care coordination first. Patient satisfaction with coordination and the number and type of recommendations are examples of research outcome measures that can show the direct outputs of care coordination and thus the effect on primary consequences of care fragmentation.

Another option to reduce the research burden for the healthcare professionals is to collect research data using health information from regular clinical care activities. Creating a structured overview of a patient's medical history, medication and writing down recommendations are regular clinical care activities, registered in the EHR for administrative purposes. Collecting research data from the EHR, after regular clinical care activities, might reduce the barrier for healthcare professionals to participate in the intervention. Another option is to ask patients to fill out questionnaires, preferably online, before consultations. This can reduce the administrative burden for healthcare professionals and leave more time to discuss the results during the consultation. Online questionnaires can also be an option to collect patient-reported outcome measures that are not directly used for clinical care.

### Strengths and limitations

One of the strengths was the practice-based participatory research design. This type of research is getting more attention as it allows an in-depth perspective. The Framework for Developing and Evaluating Complex Interventions and the Logical Framework Approach supported the development of a programme theory. By using this programme theory for the intervention, we were able to provide insights into the activities, specific outputs and outcomes, based on the theory of the mechanisms of the intervention, instead of leaving them a ‘black box’.

The measurement and description of the number and type of recommendations offer an insight into the direct outputs of the intervention and thus the potential effect on primary consequences of fragmented care. Another strength was the combination of quantitative and qualitative measures. The qualitative measures of the participant's expectations and experiences give more nuanced information, especially in patients reporting little to no improvement on the questionnaire, yet describing the potentially relevant impact of the intervention in the open questions. This nuance shows that outputs and outcomes are dependent on the individual's situation and expectations, and experienced consequences of fragmented care.

Moreover, one limitation was that we, due to COVID-19, did not reach the aimed inclusion numbers and that we adapted follow-up. Nevertheless, the number of participants and collected data were sufficient to evaluate the pilot, identify key uncertainties and learn lessons for refinement. The short follow-up time prevented insights into the effects on long-term outcomes, such as healthcare utilization.

### Implications for future research

The developed hospital care coordination team intervention resulted in mixed outputs and short-term outcomes. Recruitment and selection should be refined to include patients with multimorbidity who might benefit most, such as those who are experiencing negative consequences of care fragmentation. Outcomes of research and clinical care should align as much as possible and focus on evaluating the direct outputs of care coordination first, i.e. the number and type of recommendations and patients’ experienced effects, to show the effect of care coordination on primary consequences of care fragmentation.

## Supplemental Material

sj-docx-1-chi-10.1177_17423953231196611 - Supplemental material for A hospital care coordination team intervention for patients with multimorbidity: A practice-based, participatory pilot study Supplemental material, sj-docx-1-chi-10.1177_17423953231196611 for A hospital care coordination team intervention for patients with multimorbidity: A practice-based, participatory pilot study by Marlies Verhoeff, Janke F. de Groot, Hanneke Peters-Siskens, Erik van Kan, Yolande Vermeeren and Barbara C. van Munster in Chronic Illness
