# Supplementary material for: A hospital care coordination team intervention for patients with multimorbidity: A practice-based, participatory pilot study
Source: Chronic Illn. 2023 Sep 6;21(1):25–41. doi: 10.1177/17423953231196611 (PMC11969876; doi:10.1177/17423953231196611)
Supplement: sj-docx-1-chi-10.1177_17423953231196611 - Supplemental material for A hospital care coordination team intervention for patients with multimorbidity: A practice-based, participatory pilot study [file sj-docx-1-chi-10.1177_17423953231196611.docx]

**Supplementary file 1. Elaborate description of the hospital care coordination team intervention**

The intervention consisted of an intake appointment, a comprehensive review by a care coordination team and consultation with other healthcare providers if applicable, a follow-up consultation to discuss the results and the personalized care overview with the participant and two follow-up appointments.

*Preparation intake*

Eligible participants were invited to participate with an invitation letter. After at least one week, a research nurse contacted the eligible participants by phone and planned an intake appointment if they were willing to participate. If they refused to participate, the research nurse registered the reason of refusal if possible.
Prior to the intake, the participants received the intake questionnaire and informed consent by mail. The research physician and nurse practitioner prepared for the intake by collecting information from the EHR about the participant’s medical history, medication, medical specialties involved and healthcare utilization in the previous 12 months. We collected information about the patients’ healthcare utilisation in the 12 months prior to the intake (number of hospital admissions, hospitalization days, number of emergency department visits and outpatient visits).

*Intake appointment*

During the intake appointment, the research physician or nurse practitioner filled out and discussed the intake questionnaire with the participant. The intake questionnaire contained questions about demographics (marital status, working and living situation, etc.) and main intervention parameters: quality of life measured with the EQ5D-5L and the EQ5D-VAS (1), health literacy with the SBS-Q (2), patient activation measured with the Patient Activation Measurement 13 (PAM-13) tool (3) and patient satisfaction with care measured by using the Patient Assessment of Chronic Illness Care (PACIC) (4). Moreover, functional disability with the KATZ-ADL 6 (5) and number of medical symptoms using an adapted version of the symptoms from the Rotterdam Symptom Checklist (6). A pharmacy technician performed a medication reconciliation according to the institute’s standard procedure.

For baseline characteristics, the questionnaire contained a mental health screening measured with the Patient Health Questionnaire 2 (PHQ-2) (7) and a cognitive screening with the 6 Item Cognitive Impairment Test (6-CIT) that the research physician or nurse practitioner did during the intake(8). If present, the informal caregiver was asked to fill out the caregiver strain index. (9) Moreover, the number of applicable medication START (Screening Tool to Alert to Right Treatment) / STOPP (Screening Tool of Older Person’s Prescriptions) criteria (18) as well as the Medication Appropriateness Index (30) was determined at the time of medication reconciliation and after preparation of the final individualized care coordination plan.

Lastly, it contained three open-ended questions:

- What is most important to you at this moment (regarding the treatment of your diseases)?
- If care organization in the hospital was improved, I would like to see: …….
- In your opinion, is there a care professional who takes the lead in the coordination your care? If there is, who is this and why do you say so? If not, who do you think it should that be?

*Comprehensive review by a care coordination team*The participant’s information was presented in a care coordination team meeting, who performed a comprehensive review. A comprehensive review consisted of a discussion of the current medication, delivered hospital care and the participant’s own question (if applicable). The internist, geriatrician and hospital pharmacists discussed the indication for medication and follow-up, and if unclear, the research physician or nurse practitioner would contact the prescribing medical specialist to clarify the indication. If applicable, the care coordination team gave advice on possible medication changes or changes that possibly could be made by involved healthcare professionals. The team could also give advice on other healthcare related matters that emerged during the intake or review process. The advice was recorded on a web-based data management platform (Castor). After the team meeting, the research physician or nurse practitioner drafted up the personalized care overview, containing the medical history, medication, involved medical specialists and the advice by the care coordination team. The medical history was also translated in layman’s termsThe research physician or nurse practitioner also contacted the general practitioner, to discuss the results of the comprehensive review and the team’s advice.

*Result consultation to discuss the results*The results of the comprehensive review and the personalized care overview were discussed during a result consultation. The nurse practitioner and research physician discussed the advice by the care coordination team and recorded any remarks or new information that emerged from the consultation.

*Follow-up per protocol: at 4-6 weeks and at 3 months after the intake*After 4-6 weeks, the nurse practitioner or research physician phoned the participant to check whether the participant had any questions about the personalized care overview or the advices. Any remarks were recorded. At 3 months after the intake, participants had another follow-up consultation (in real live, or during to COVID-19 pandemic by phone). Any remarks by the participant concerning the individualized care overview or advices were recorded. Moreover, the research physician or nurse practitioner filled out the follow-up questionnaire with the participant. This follow-up contained the study parameters for patient satisfaction, quality of life, patient activation from the intake questionnaire, but also two open-ended questions where participants were asked to reflect on their prior answers and whether the intervention contributed.

*Adjusted result consultation and/or follow-up due to COVID-19 crisis*Six months after starting the inclusion, the COVID-19 crisis started. Due to the national and local measures, it was not possible to continue the intervention and follow-up per protocol. All new intakes were cancelled. Participants who had already had an intake, received an adjusted result consultation or adjusted follow-up by phone.

**References**

1. M. Versteegh M, M. Vermeulen K, M. A. A. Evers S, de Wit GA, Prenger R, A. Stolk E. Dutch Tariff for the Five-Level Version of EQ-5D. Value Health. 2016 Jun;19(4):343–52.

2. Fransen MP, Van Schaik TM, Twickler TB, Essink-Bot ML. Applicability of Internationally Available Health Literacy Measures in the Netherlands. J Health Commun. 2011 Sep 30;16(sup3):134–49.

3. Rademakers J, Nijman J, van der Hoek L, Heijmans M, Rijken M. Measuring patient activation in the Netherlands: translation and validation of the American short form Patient Activation Measure (PAM13). BMC Public Health. 2012 Dec;12(1):577.

4. Vrijhoef HJM, Berbee R, Wagner EH, Steuten LMG. Quality of integrated chronic care measured by patient survey: identification, selection and application of most appropriate instruments. Health Expect. 2009 Dec;12(4):417–29.

5. Reijneveld SA, Spijker J, Dijkshoorn H. Katz’ ADL index assessed functional performance of Turkish, Moroccan, and Dutch elderly. J Clin Epidemiol. 2007 Apr;60(4):382–8.

6. de Haes J, van Knippenberg F, Neijt J. Measuring psychological and physical distress in cancer patients: structure and application of the Rotterdam Symptom Checklist. Br J Cancer. 1990 Dec;62(6):1034–8.

7. Kroenke K, Spitzer RL, Williams JBW. The Patient Health Questionnaire-2: Validity of a Two-Item Depression Screener. Med Care. 2003 Nov;41(11):1284–92.

8. Tuijl JP, Scholte EM, Craen AJM, Mast RC. Screening for cognitive impairment in older general hospital patients: comparison of the Six-Item Cognitive Impairment Test with the Mini-Mental State Examination: 6CIT cognitive impairment test. Int J Geriatr Psychiatry. 2012 Jul;27(7):755–62.

9. van Exel NJA, Scholte op Reimer WJM, Brouwer WBF, van den Berg B, Koopmanschap MA, van den Bos GAM. Instruments for assessing the burden of informal caregiving for stroke patients in clinical practice: a comparison of CSI, CRA, SCQ and self-rated burden. Clin Rehabil. 2004 Mar;18(2):203–14.

**
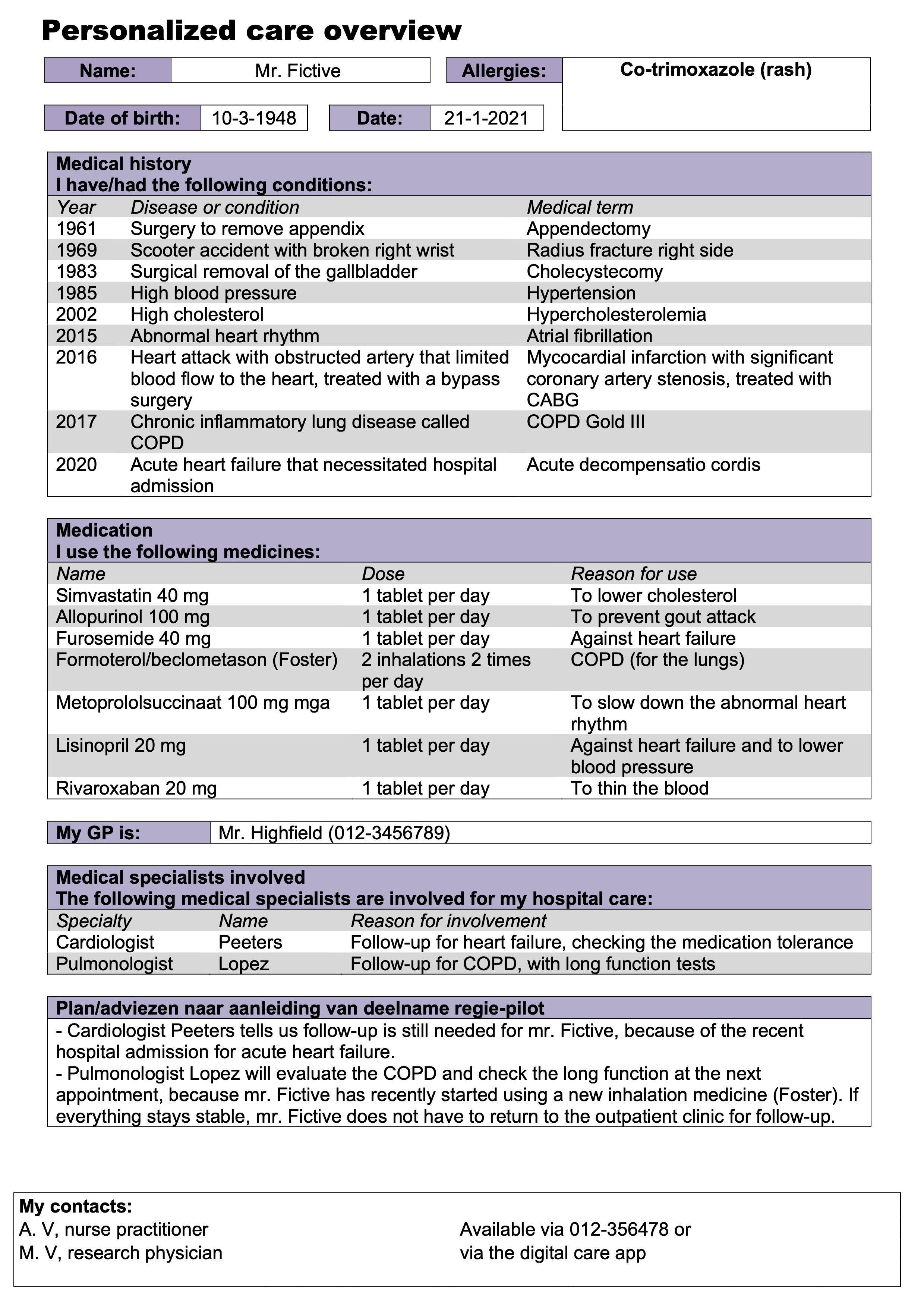
Supplementary file 2. Example of a personalized care overview Supplementary file 3. Comparison of characteristics of patients willing to participate and patient who declined participation**

| **Characteristic** | **Willing to participate (n=40)** | **Refused participation (n=90)** | **p-value** |
| --- | --- | --- | --- |
| Age, years, median (IQR) | 73.4 (63.9-77.4) | 73.6 (69.3-79.9) | 0.25 |
| Sex, n (%) female | 23 (58) | 53 (59) | 1 |
| Number of involved medical specialties in 2018, median (IQR) | 7 (7-8) | 7 (7-8) | 0.33 |

**Supplementary file 4. Categorized answers to three open questions at intake**

| **Q1. What is most important to you at this moment (regarding the treatment of your diseases)?** | **Q2. If care organization in the hospital was improved, I would like to see:** | **Q3. In your opinion, is there a care professional who takes the lead in the coordination of your care? If yes, who is this and why do you say so? If no, who do you think it should be?** |
| --- | --- | --- |
| - To improve or maintain (independent) daily functioning (10x) - To decrease pain or keep it under control (8x) - To be taken seriously, also with complaints outside the specialism (3x) - Getting the right treatment (3x) - Keep diseases under control (3x) - Feel better or less tired; improvement (3x) - Collaboration and coordination between specialists (2x) - Continuation of current care (1x) - Rest, medication and keep track of lifestyle (1x) - I have accepted my diseases (1x) - To be able to see my own health record (1x) - To keep having fun in my life (1x) - That I will not break down, am able to care for my partner and do not burden my children (1x) - That therapy is covered by insurance (1x) | Coordination and overview:   - More communication and collaboration between medical specialists (4x) - To have 1 coordinating physician who has periodical multidisciplinary meetings or keeps track of your follow-up (2x) - That patients are seen as a whole (2x) - That there is a medication overview (2x) - All appointments in one day (1x) - Better availability and a contact person (1x)   General organization of care:   - Hospital care is organized adequately (8x) - Better work environment and less pressure for nurses (3x) - Electronic systems to support patients and access to your electronic health record (1x) - That appointments start on time (1x)   Getting the right treatment:   - That doctors take complaints seriously (2x) - That both my physical and my mental conditions are treated at once, in one admission (1x) - To find the right specialist for my treatment (1x) - That my therapy is arranged for me (1x) - Don’t know (2x) | - Yes there is (10x)   - GP (2x)   - GP and medical specialist (2x)   - medical specialist (4x)   - home care (2x) - No care professional currently coordinating care (18x)   - informal care giver (2x)   Which professional should coordinate care:   - don’t know who could (9x) - someone from administration or from a general department (2x) - GP (2x) - home care (2x) - GP or hematologist (1x) - pulmonologist (1x) - someone in hospital who knows about every discipline (1x) |
